# Exonize: a tool for finding and classifying exon duplications in annotated genomes

**DOI:** 10.1093/bioadv/vbaf177

**Published:** 2025-07-28

**Authors:** Marina Herrera Sarrias, Christopher W Wheat, Liam M Longo, Lars Arvestad

**Affiliations:** Department of Mathematics, Stockholm University, Stockholm, SE-106 91, Sweden; Swedish e-Science Research Centre, Stockholm, SE-100 44, Sweden; Department of Zoology, Stockholm University, Stockholm, SE-106 91, Sweden; Earth-Life Science Institute, Institute of Science Tokyo, Tokyo 152-8550, Japan; Blue Marble Space Institute of Science, Seattle, Washington 98104, United States; Department of Mathematics, Stockholm University, Stockholm, SE-106 91, Sweden; Swedish e-Science Research Centre, Stockholm, SE-100 44, Sweden

## Abstract

**Summary:**

The protein-coding regions of eukaryotic genes are fragmented into exons that, like the genes within which they are situated, can be duplicated, deleted, or reorganized. Cataloging and organizing within-gene exon similarities is necessary for a systematic study of exon evolution and its consequences. To facilitate the study of exon duplications, we present Exonize, a computational tool that identifies and classifies coding exon duplications in annotated genomes. Exonize implements a graph-based framework to handle clusters of related exons resulting from repeated rounds of exon duplication. The interdependence between duplicated exons or groups of exons across transcripts is classified. By identifying duplication events between exonic and intronic regions, Exonize can detect unannotated or degenerate exons. To aid in data parsing and downstream analysis, the Python module exonize_analysis is provided. The application of Exonize to 20 eukaryote genomes identifies full-exon duplications in at least 4% of vertebrate genes, with more than 900 human genes having a full-exon duplication event.

**Availability and implementation:**

Exonize is available at https://github.com/msarrias/exonize.

## Introduction

Sequence repetition is a key driver of protein evolution, from fragment duplication in the structural evolution of symmetric protein folds (Eck and Dayhoff 1966) to gene duplication as a precursor to functional divergence (Ohno 1970). Upon the emergence of exons, which can include protein-coding regions (Aspden *et al.* 2023), a new form of evolution by repetition became possible: exon duplication (Letunic et al. 2002, Ivanov and Pervouchine 2022). Previous studies on exon duplication dynamics (Letunic *et al.* 2002, Ivanov and Pervouchine 2022, Martinez Gomez *et al.* 2021, 2022) have established exon duplication as a common evolutionary process in eukaryotes. However, at present, there is no accessible and user-friendly tool for identifying exon duplications. Although alignment tools such as Exonerate (Slater and Birney 2005) and GMAP (Wu and Watanabe 2005) are widely used for modeling gene structure, they do not provide dedicated functionality for detecting and classifying exon duplication events. If the evolutionary consequences of exon duplications are to be fully understood, tools to identify and classify exon duplication events must be readily available. To this end, we introduce Exonize, a tool for finding and classifying coding exon duplications in annotated genomes.

The design of Exonize centers on three goals: Foremost, Exonize identifies and groups duplicated exons within protein-coding regions, i.e. protein-coding exons, hereafter referred to as *exons* for brevity. Next, Exonize classifies the relationship between duplication events with respect to exonic and intronic boundaries within a gene, potentially identifying unannotated or degenerate (pseudo) exons. Finally, Exonize classifies the interdependence between duplicates or groups of duplicates across transcripts. Exonize facilitates comparisons between multiple species and allows a systematic assessment of the influence of repeat masking.

Following a detailed description of the implementation of Exonize, we demonstrate that it detects nearly all exon duplication events in a simulated dataset and most of the events in the manually curated dataset presented in (Martinez Gomez *et al.* 2021). Application of Exonize to 20 eukaryotic genomes reveals that ∼4%–7% of vertebrate genes contain at least one full coding exon duplication and identifies hundreds of putative exon duplicates within each genome.

## Overview

Exonize requires a genome assembly in FASTA format and corresponding gene annotations in GFF or GTF format. Only protein-coding exons, regions annotated as coding sequences (CDS), are considered in the search. As annotated exon boundaries can overlap or be nested, a set of representative exons is chosen for each gene. Since exons can have sequence similarity unrelated to an exon duplication process, as in repetitive protein folds, length similarity is required.

**Figure 1.**
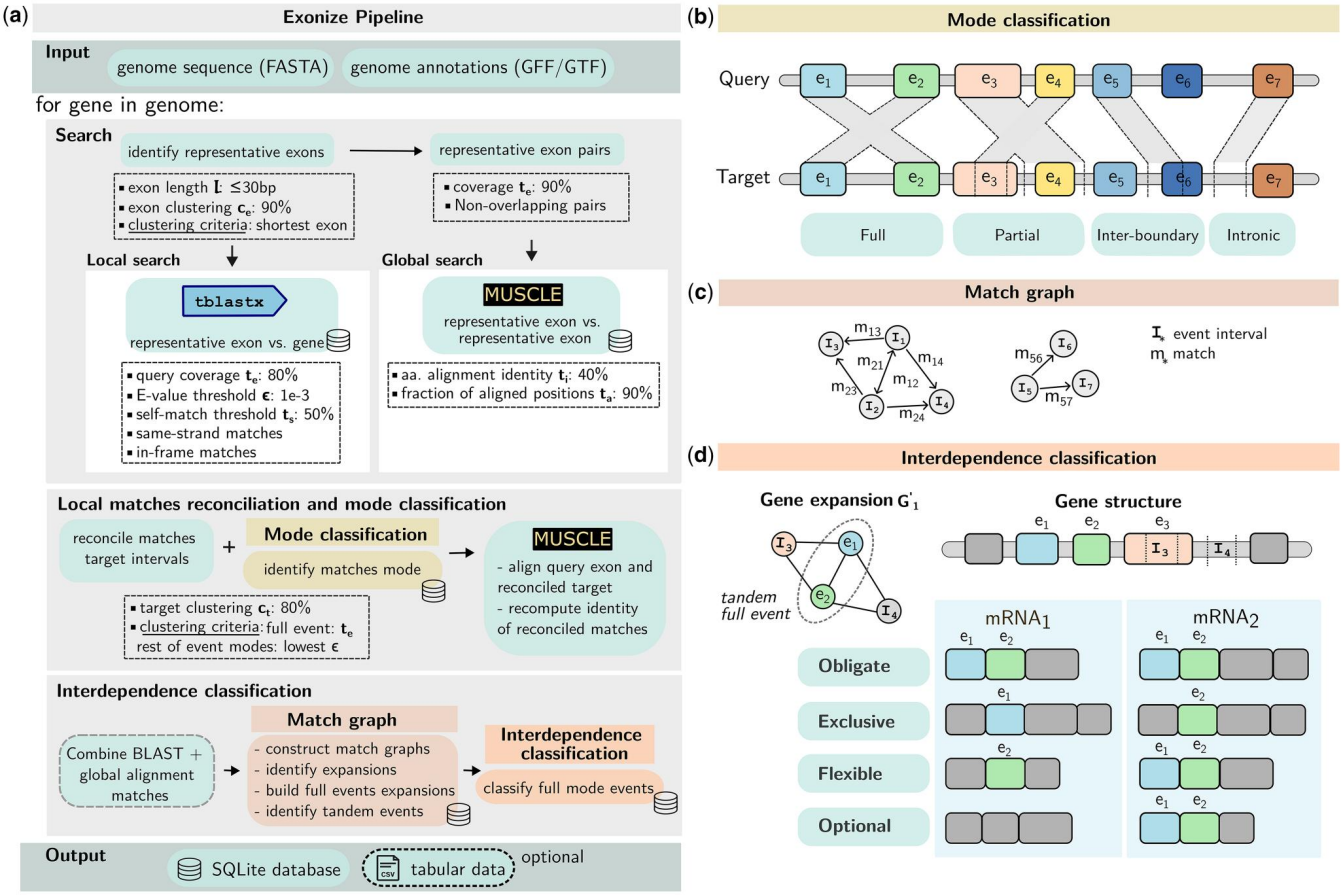
(a) The Exonize pipeline, with default parameters and search criteria indicated in dotted boxes. The database icon denotes data available in the output database. (b) Illustration of the match classification system. Note that the query and the target genes are the same gene. Boxes indicate exons, and shading indicates matches. Self-hits are not shown for clarity. (c) A directed match graph for a gene with two exon duplications (two expansions). (d) Transcript interdependence classification for a gene with two transcripts (mRNA_1_ and mRNA_2_) and two tandem exon duplicates, $e_{1}$ and $e_{2}$. $I_{3}$ represents a partial duplication event, and $I_{4}$ illustrates an intronic match and is thus excluded from interdependence classification.

We define a *full-exon duplication* as a protein-coding exon that has been duplicated in its entirety within the boundaries of its parent gene. Exonize detects full-exon duplications using global and local alignment methods. The global approach identifies alignments between representative exons of similar length using Muscle5 (Edgar 2022) and is subject to amino acid identity and coverage cutoffs. The local approach complements the global search by querying representative exons against the entire gene, including introns, using tblastx (Camacho *et al.* 2009) and is subject to E-value and coverage cutoffs. Regions of alignment found by both methods are referred to as *matches*. Users have the option to restrict the search to a single method or combine results from both methods. An overview of Exonize is provided in Fig. 1A.

### Representative exon selection

A gene *g* is defined here as a collection of transcripts consisting of a set of exons defined as closed intervals of the form [*a*, *b*], where *a* and *b* indicate the start and end genomic coordinates, respectively. Accounting for all exon coordinates, however, can result in many near-equivalent duplication events due to overlapping coordinates between exons. Thus, Exonize restricts the search to a *set of representative exons* E. Each representative exon in E must be longer than the nucleotide length threshold *l* and no pair of representative exons overlap more than the fractional overlap cutoff $c_{e}$. The length criterion *l* is intended to exclude false positives from short, repetitive regions. Setting $c_{e}=0$ restricts E to entirely non-overlapping exons. Setting $c_{e}=1$ admits all exons longer than *l* to E.

To generate E, the set of exons from all transcripts in *g* are clustered according to Supplementary Algorithm S1. The fractional overlap between exons is calculated relative to the length of the longer exon (Definition 1). For each cluster of overlapping exons, the shortest exon is taken to be the representative, maximizing alignment coverage during the search step. To ensure identical cluster formation between runs, intervals are sorted first by the lower boundary and then by the upper boundary.

Definition 1. For any pair of closed intervals $I_{i},I_{j}\in I$, where I refers to the set of closed intervals in the natural domain, the minimum interval overlap ratio O:I×I→[0,1] is defined as:

(1) $$O(I_{i},I_{j})=>\{\begin{array}{cc} \frac{\ell (I_{i}\cap I_{j})}{\max(\ell (I_{i}),\;\ell (I_{j}))} & \text{if}\;I_{i}\cap I_{j}\neq \emptyset ,\\ 0 & \text{otherwise} \end{array}$$

where ℓ([a,b])=b−a+1 is the length of the interval.

### Finding putative exon duplications

Exonize queries sequence similarity between representative exons and the genes within which they are situated using tblastx (Camacho *et al.* 2009). Accepted regions of sequence similarity from local searching, or matches, must satisfy an E-value cutoff ϵ, taken from the pairwise search, and a query coverage cutoff $t_{e}$, where $t_{e}$ denotes the minimum fraction of the query exon (a representative exon) that must be aligned. Matches where the fractional overlap of the query exon to itself is greater than or equal to $t_{s}$, the self-hit threshold, are considered self-matches and are ignored. The query coverage cutoff $t_{e}$ determines how a match is classified (Fig. 1B): A match is considered **full-length** if the query exon overlaps at least a fraction $t_{e}$ of both its length and the length of an annotated exon (not necessarily a representative exon). A **partial** match occurs when either the query exon matches within an annotated exon or completely encompasses an annotated exon. In either case, $t_{e}$ is only satisfied for either the query exon or the annotated exon. An **inter-boundary** match occurs when the alignment extends beyond $t_{e}$ of the length of the query exon but less than $t_{e}$ of the annotated exon length and encompasses both exonic and intronic regions. Finally, an **intronic** match occurs when a query exon aligns to an intronic region by more than $t_{e}$.

Lower values of $t_{e}$ yield more matches classified as full-length, whereas higher values of $t_{e}$ yield more matches classified as partial. Based on the above criteria, full-length matches (which correspond to putative full-exon duplication events) are always reciprocal, whereas all other match types are non-reciprocal. As with representative exons, overlaps between matches are possible.

### Reconciliation of target intervals

Let *M* represent the set of matches associated with *g*, where each match m∈M is a pair of closed intervals of the form $m=(I_{q},I_{t})$, with $I_{q}$ and $I_{t}$ corresponding to the query and target genomic intervals, respectively. To address overlaps between distinct targets within the same genomic regions, a *reconciled target* $I_{t}^{\prime }$ of the target interval $I_{t}$ is introduced. The reconciled intervals are found by clustering the target intervals using Supplementary Algorithm S1, subject to an overlap threshold $c_{t}$, and selecting a representative interval from the cluster. Clusters composed of full-length matches are reconciled against the annotated exon coordinates that map to the target coordinates. Other clusters are reconciled against the target coordinates of the most significant match within the cluster. To update the target reading frame, the three translated target sequences are aligned pairwise to the translated query exon sequence (for which the reading frame is known) using Muscle5 (Edgar 2022). The reading frame with the highest alignment identity is selected. Reconciled matches may overlap.

### Cases of full-exon duplications

The global search is restricted to identifying full-length exon duplication events. In this approach, pairs of representative exons are aligned using Muscle5 (Edgar 2022) if the ratio of the shorter exon to the longer exon is greater than $t_{p}$. Alignments are performed on both DNA and protein sequences. Matches are retained in *M* if the protein alignment identity satisfies the cutoff $t_{i}$ and the fraction of aligned positions satisfies the cutoff $t_{a}$. The global alignment approach allows for more sensitive detection of full-length exon duplications that may have been missed by the local search. In the event that a full-exon duplication event is classified as partial by the local search but satisfies the global requirements here, it is taken to be a full-length duplication event.

### Identification of exon families with match graphs

Within a gene, multiple rounds of exon duplication can result in families of related exons, which Exonize handles using a graph-based approach. Let $D=(V,\vec{E})$ be a directed graph encoding the union of global and local matches *M* associated with *g*. The set of vertices *V* consists of the intervals of the representative exons and reconciled targets. The set of ordered edges $\vec{E}$ corresponds to the matches themselves. For local matches, the natural notion of order in $\vec{E}$ derives from the query-to-target directed relationship. Matches detected through the global search are considered reciprocal and are therefore represented with bidirectional edges. Two distinct vertices $I_{i},I_{j}^{\prime }\in V$ are said to be connected if there exists a match $m_{ij}=(I_{i},I_{j}^{\prime })\in \vec{E}$ directed from the query $I_{i}$ to the target $I_{j}^{\prime }$. To resolve reciprocal matches, we consider the undirected version $G=(V,E^{\prime })$ of *D*. In this case, *G* may form a single connected component or be composed of multiple connected subgraphs. We refer to these subgraphs as *expansions* because they arise from repeated exon duplication events. The count of expansions *n* estimates the number of distinct exons in *g* that have been duplicated, and the size of each expansion, denoted as *k*, relates to the number of duplication events within an expansion, taken to be k−1 (i.e. assuming that no multi-exon duplication occurred). Additionally, we define a *full-length expansion* as an expansion composed only of full-length matches. Figure 1C illustrates the match graph for a gene with two expansions.

### Tandemness of exon duplication events

A pair of exons is said to be tandem if they are adjacent along the gene sequence; that is, if no complete exon lies between them. Similarly, the events within a full-length expansion are considered tandem if they involve exons that form a sequence of adjacent exon duplications. We assess the adjacency of the events within a full-length expansion by first sorting the events based on the start and end coordinates. We then construct a sequence of predecessor-successor pairs and categorize them based on whether they are tandem or not. This is done by clustering the set of coding exons associated with the gene using Supplementary Algorithm S1 with an overlapping cutoff of 0 and verifying whether the predecessor and successor coordinates in each pair belong to consecutive clusters.

### Exon duplication interdependence across transcripts

A duplication event can be classified based on its interdependence across the transcripts of *g* (Fig. 1D). The **obligate** scenario occurs when both duplicates are found within all transcripts; the **exclusive** case occurs when no transcript contains both duplicates; the **flexible** case occurs when each transcript includes at least one duplicate but without showing an exclusive or obligate pattern. Lastly, duplicates are classified as **optional** when there is at least one transcript that lacks both duplicates. Optional cases can be further subdivided into obligate, exclusive, or flexible depending on the relationships between the duplicates in the transcripts in which they are present. This classification scheme extends to groups of duplicates as well, as in the case of full-length expansions.

## Configuration

Program usage and available options are detailed in the user manual. Exonize outputs an SQLite3 database, which provides type-safe and efficient post-processing. The structure of the database is illustrated in Supplementary Figure S1. In addition, users have the option to export a simplified version of the output in CSV format.

## Implementation and computational aspects

Exonize is an open-source command-line tool available for installation via the Python Package Index (PyPI.org). The source code, the user manual, a sample dataset, and a tutorial on how to use the exonize_analysis module are all accessible on GitHub. To run Exonize, local installations of BLAST+ (Camacho *et al.* 2009), Muscle5 (Edgar 2022), and SQLite are required. Parallel processing is used to speed up the search and reconciliation steps.

## Validation

The performance of Exonize was validated using both simulated data and a manually curated dataset of exon duplications in the human genome (GENCODE version 33) (Martinez Gomez *et al.* 2021). Simulated exon duplication events were inserted into the human Y chromosome (Supplementary Section 3.1). Each simulated dataset included 445 full-length exon duplication events and 420 intronic events. Five datasets were constructed to cover a range of evolutionary distances, from 0.2 to 2.0 expected mutations per site. For full-length duplications, Exonize recovered 97% of events, even at high evolutionary distances. The majority of intronic events, 84% in total, were also recovered. The missed intronic events were mainly due to the performance of BLAST+ (Camacho *et al.* 2009) (see Supplementary Figure S2). Among the 174 full-length exon duplication events in the human genome proposed in (Martinez Gomez *et al.* 2021), Exonize successfully recovered 64% of events in total (Supplementary Figure S3) and 98% of events that were in principle detectable based on the amino acid identity cutoff used. Although a less stringent search recovered 86% of events, it was accompanied by a large increase in likely false positives. A sensitivity analysis of the local search performed on the human genome (GRCh38.p14) using different parameter settings can be found in Supplementary Table S1.

## Results

Application of Exonize to 20 unmasked eukaryote genomes (Table 1) obtained from the Ensembl database (release 114) (Dyer *et al.* 2025) reveals full-exon duplication events within ∼4%–7% of vertebrate genes. Significantly more genes were identified as having partial, inter-boundary, and intronic exon duplication events. Lower eukaryotes, on the other hand, generally had fewer duplication events, and no full-exon duplication events were detected in yeast.

**Table 1.** Exon duplication statistics for 20 eukaryotes. The analysis was performed with default parameters.

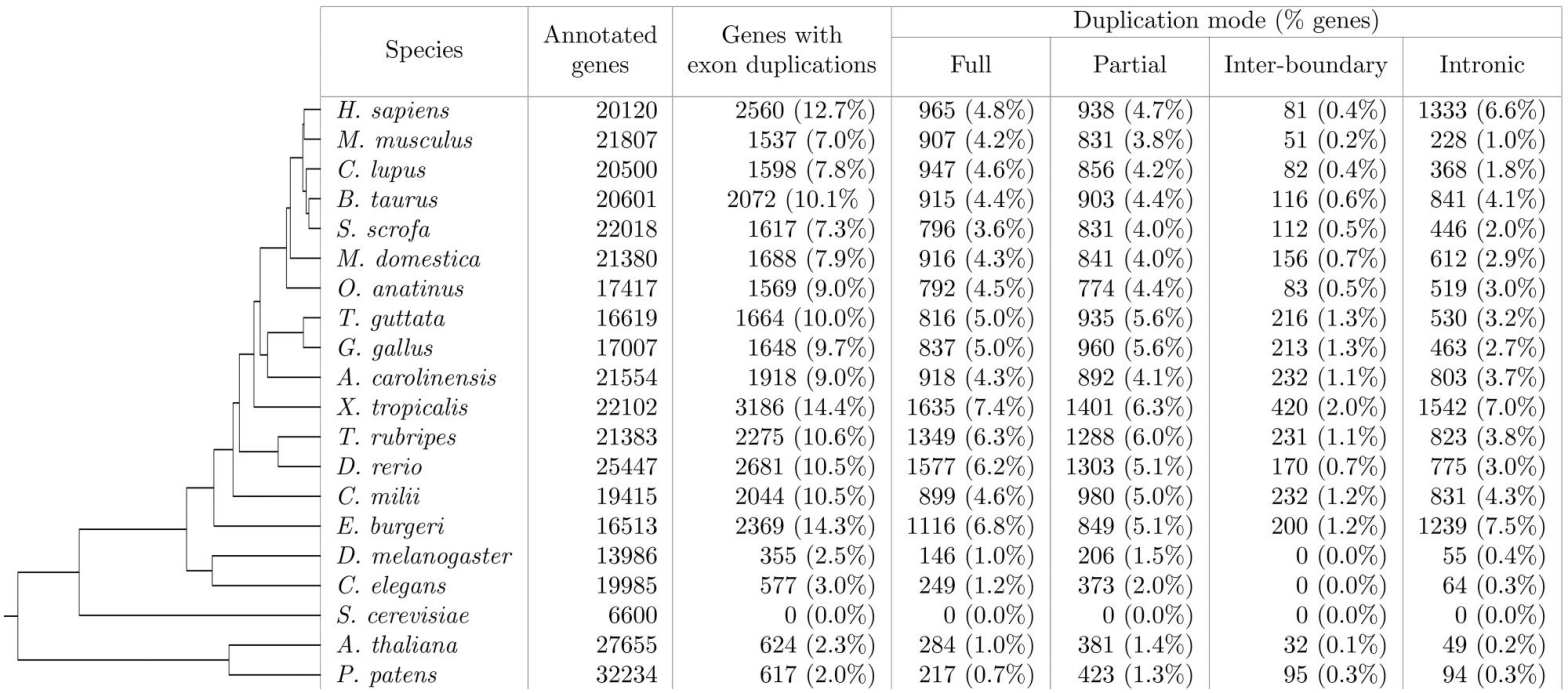

## Conclusion

We introduce Exonize, a tool for identifying and classifying exon duplications. Using a simple heuristic approach, Exonize can detect and handle redundant matches that arise from reciprocal and overlapping duplications. This tool facilitates systematic studies of exon duplications in gene and protein evolution.

## Supplementary Material

vbaf177_Supplementary_Data

## Data Availability

All data presented in this study are available at Zenodo, DOI 10.5281/zenodo.13370679 under a CC BY 4.0 license.
