# Supplementary material for: Exonize: a tool for finding and classifying exon duplications in annotated genomes
Source: Bioinform Adv. 2025 Jul 28;5(1):vbaf177. doi: 10.1093/bioadv/vbaf177 (PMC12343006; doi:10.1093/bioadv/vbaf177)
Supplement: vbaf177_Supplementary_Data [file vbaf177_supplementary_data.pdf]

Exonize Supplementary Material

1 Intervals clustering algorithm

Algorithm S1 Interval Clustering

Require: $c > 0$: overlap threshold
Require: `intervals`: sorted list of intervals

```
procedure ClusterIntvs(c, intervals[])
    clusters ← {}
    skip_intervals ← {}
    for I in intervals do
        if I not in skip_intervals then
            updated ← true
            cluster ← {I}
            while updated do
                updated ← false
                candidates ← intervals \ skip_intervals
                for J in candidates do
                    if ∀K ∈ cluster : O(J, K) ≥ c then
                        cluster ← cluster ∪{J}
                        updated ← true
                    end if
                end for
            end while
            skip_intervals ← skip_intervals ∪ cluster
            clusters ← clusters ∪ cluster
        end if
    end for
    return clusters
end procedure
```

2 Exonize Output

The output of Exonize is an SQLite3 database. Figure S1 illustrates the structure of the database. Users have the option to export a compressed directory containing individual CSV files for the main tables in the SQLite output, excluding the `Genes` and `Local_matches` tables.

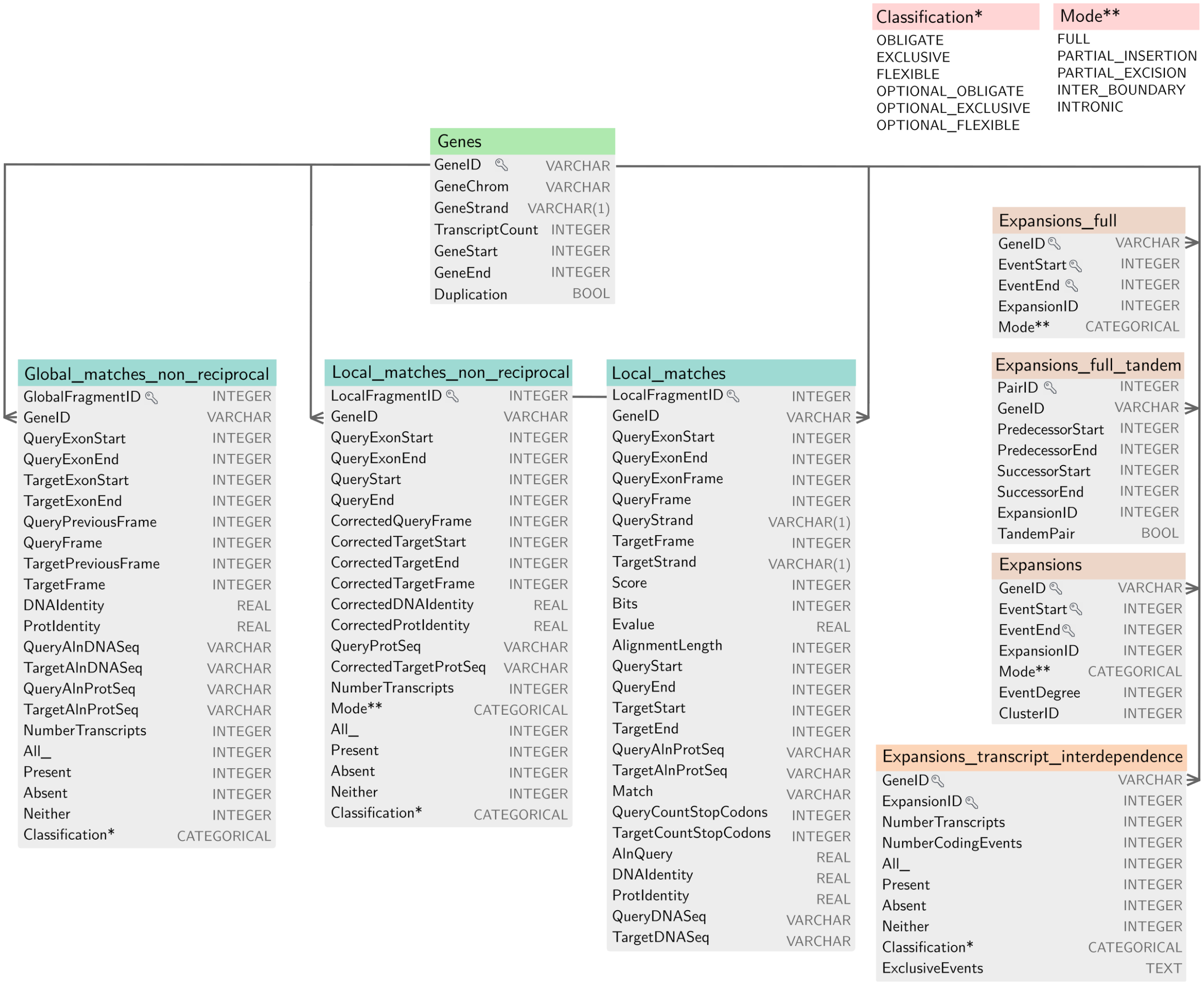

Figure S1: Exonize SQLite3 database diagram.

The tables are described as follows:

- `Genes`: All genes analyzed for exon duplications.
- `Local_matches`: All unfiltered matches identified by the local search.
- `Local_matches_non_reciprocal`: A filtered version of `Local_matches` containing non-reciprocal and reconciled local matches that meet the search criteria.
- `Global_matches_non_reciprocal`: Global matches that satisfy the search criteria.
- `Expansions`: All expansions. Each record corresponds to a node in an expansion graph constructed from local and global matches.
- `Expansions_full`: Expansion records of full-length exon duplications.
- `Expansions_full_tandem`: Pairs of consecutive full-length events within an expansion, indicating whether they are in tandem.
- `Expansions_transcript_interdependence`: Transcript interdependence classifications for expansions in the `Expansions_full` table.

3 Validation

The ability of Exonize to identify exon duplication events was evaluated using a simulated dataset and the manually curated dataset presented in (Martinez Gomez et al., 2021). The analyses were performed using Exonize with BLAST+ (Camacho et al., 2009) version 2.12.0 and Muscle5 (Edgar, 2022). The same parameter settings were used for both analyses: E-value threshold 10^{-3}, minimum exon length $l = 30$, self-hit threshold $t_s = 0.5$, exon clustering $c_e = 0.9$, target clustering $c_t = 0.8$, query coverage $t_e = 0.8$, fraction of aligned positions $t_a = 0.9$, amino acid identity threshold $t_i = 0.4$, and global pairs coverage threshold t_p=0.9.

3.1 Simulated dataset

The human Y chromosome (Ensembl v. GRCh38.p14) was used as a template. A total of 865 duplication events were generated from 449 annotated exons longer than 50 base pairs. The 449 candidate exon sequences were translated and subjected to varying degrees of amino acid substitution by the JTT model (Jones et al., 1992) using

seq-gen (Rambaut and Grassly, 1997). Evolutionary distance from the parent exon ranged from 0.2-2.0 expected amino acid mutations per site. Insertions and deletions were not considered. The amino acid sequences were then reverse-translated and randomly reinserted into intronic regions of the corresponding gene. To validate both the global and local approaches, 445 of the artificially evolved exons were annotated as protein-coding sequences (CDS) in the GFF file so that they would be selected as representative exons and included in the global search for full-length exon duplication events. The remaining 420 were annotated as intronic sequences in the GFF file and were thus detectable only by the local search. Full-length events were robustly detected across all evolutionary distances, while the detection of intronic duplicates was more sensitive to evolutionary distance (Figure S2). Nevertheless, the local search approach was able to identify more than 60% of the duplication events even at the high evolutionary distance of 2 expected amino acid mutations per site.

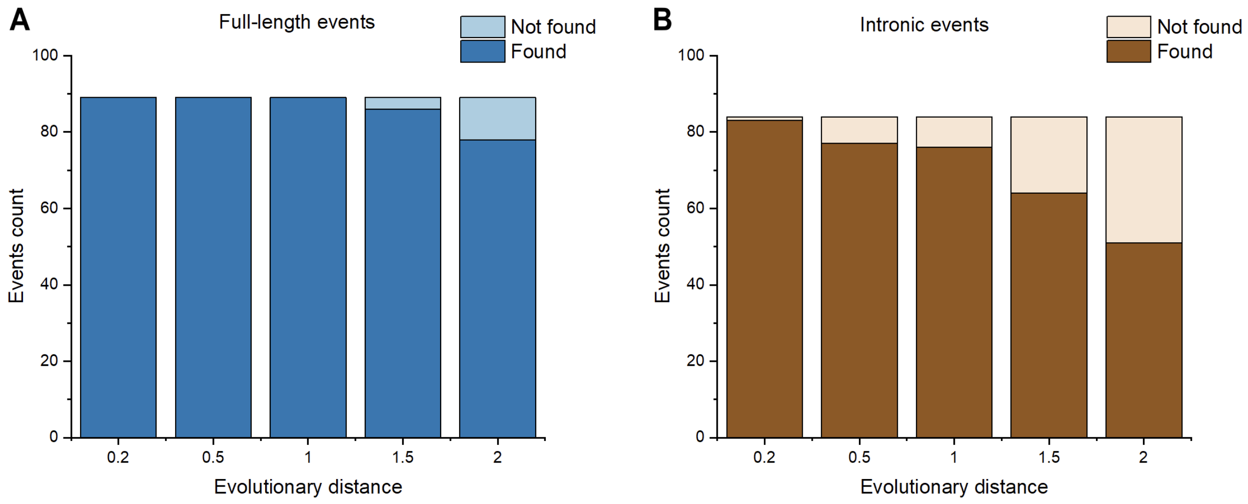

Figure S2: Recovered exon duplicates from simulated **(A)** full-length and **(B)** intronic duplication events with increasing evolutionary distance.

3.2 Human dataset

A manually curated database of human exon duplications was reported in (Martinez Gomez et al., 2021). Unlike the simulated data used for validation, natural exon duplicates vary in length. Moreover, natural exons can have overlapping and nested

structures, complicating their identification. Out of the 214 duplication events identified by (Martinez Gomez et al., 2021), 174 events satisfy the minimum coverage and length cutoffs set for validating Exonize (see above for parameter settings). The majority of manually curated exons were successfully identified by Exonize (Figure S3). Overwhelmingly, missed events are associated with low amino acid identities, highlighting the potential limitations of a sequence-only search approach. Events below the identity cutoff were detected by the local search, which is subject to an E-value cutoff instead. Relaxing the search criteria with an E-value threshold of 1, exon clustering $c_e = 1$, and amino-acid identity threshold $t_i = 0.1$ increased the true positive rate to 86%. However, it also increased the number of identified full-length exon duplication events from 947 to over 12,000 events.

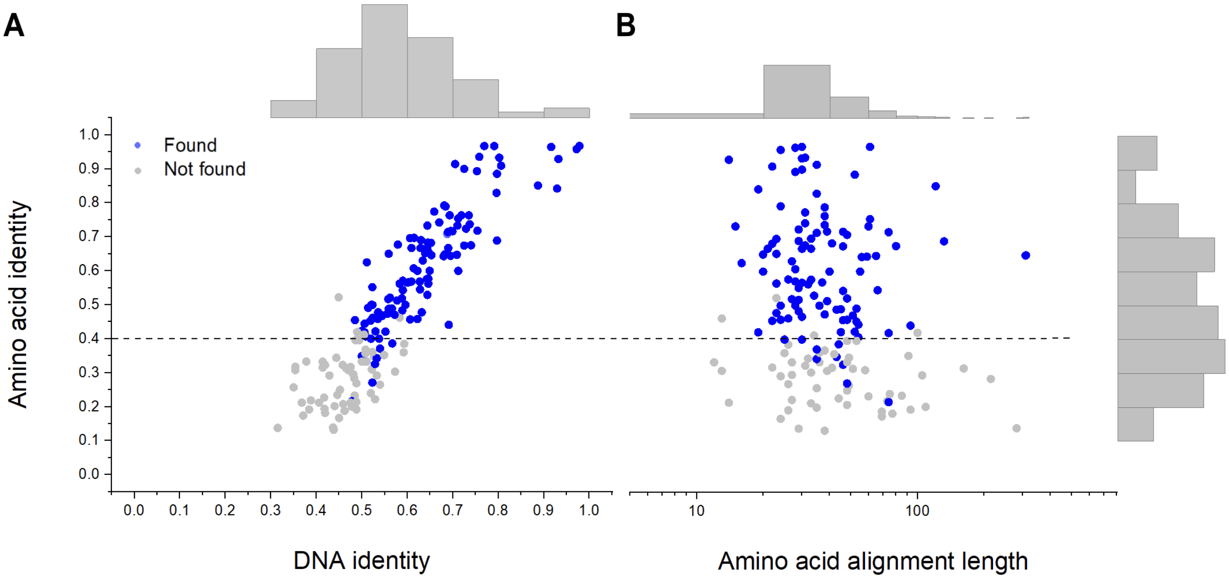

Figure S3: Exon duplicates identified by Gómez *et al.* were successful identified (blue) by Exonize across a range of pairwise identities **(A)** and alignment lengths **(B)**. The dotted black line indicates the amino acid identity threshold used in the global search, below which duplicate detection is rare and due to the local search.

4 Local search sensitivity analysis

Table S1 reports a breakdown of the duplication mode (at the match and gene level) and the percentage of overlapping matches under different local search configurations. In general, a higher clustering exon threshold increased the inclusion of overlapping exons in the set of representative exons, leading to more overlapping matches. Lower values of the clustering match threshold yielded more reconciliation of overlapping matches. The classification of matches as full or partial events depends on the stringency of the query coverage parameter: less stringent searches yield more matches classified as full, while more stringent searches result in more partial classifications.

Parameters			Overlapping duplicates	Duplicates mode distribution (% reconciled duplicates)			
Clustering exon threshold c_e	Query coverage t_e	Clustering matches threshold c_t		Full	Partial	Inter-boundary	Intronic
90%	100%	100%	211 (11.7%)	778 (43.1%)	282 (15.6%)	12 (0.7%)	733 (40.6%)
90%	70%	70%	7541 (21.8%)	9752 (28.2%)	5058 (14.6%)	74 (0.2%)	19711 (57.0%)
90%	80%	80%	7643 (26.1%)	8329 (28.5%)	5089 (17.4%)	95 (0.3%)	15735 (53.8%)
90%	90%	90%	5895 (28.8%)	6515 (31.8%)	4097 (20.0%)	118 (0.6%)	9760 (47.6%)
70%	90%	90%	5439 (27.0%)	6431 (31.9%)	4052 (20.1%)	115 (0.6%)	9550 (47.4%)
0%	90%	90%	4921 (25.2%)	6246 (32.0%)	3911 (20.0%)	107 (0.5%)	9290 (47.5%)
100%	90%	90%	6079 (29.4%)	6607 (32.0%)	4126 (20.0%)	120 (0.6%)	9812 (47.5%)

(**A**) Mode distribution and percent overlap of reconciled exon duplication matches. Percentages are calculated relative to the total number of matches.

Parameters			Duplication mode (% genes)				
Clustering exon threshold c_e	Query coverage t_e	Clustering matches threshold c_t	All	Full	Partial	Inter-boundary	Intronic
90%	100%	100%	333 (1.7%)	70 (0.3%)	92 (0.5%)	11 (0.1%)	203 (1.0%)
90%	70%	70%	2742 (13.6%)	975 (4.8%)	1055 (5.2%)	62 (0.3%)	1495 (7.4%)
90%	80%	80%	2407 (12.0%)	756 (3.8%)	945 (4.7%)	81 (0.4%)	1333 (6.6%)
90%	90%	90%	1942 (9.7%)	514 (2.6%)	721 (3.6%)	95 (0.5%)	1108 (5.5%)
70%	90%	90%	1936 (9.6%)	509 (2.5%)	712 (3.5%)	93 (0.5%)	1103 (5.5%)
0%	90%	90%	1897 (9.4%)	498 (2.5%)	683 (3.4%)	86 (0.4%)	1092 (5.4%)
100%	90%	90%	1945 (9.7%)	516 (2.6%)	725 (3.6%)	97 (0.5%)	1112 (5.5%)

(**B**) Percentage of genes with duplications for the different modes. Percentages are computed relative to the total number of protein-coding genes.

Table S1: Exonize local search sensitivity analysis performed on the Ensembl (release 114), unmasked version of the human genome with E-value threshold 10^{-3}, minimum exon length $l = 30$, self-hit threshold $t_s = 0,5$ and different parameters settings for the exon clustering c_e, target clustering c_t, and query coverage t_e thresholds. Default values are highlighted in dark gray.

References

Christiam Camacho, George Coulouris, Vahram Avagyan, et al. BLAST+: architecture and applications. *BMC Bioinformatics*, 10:1–9, 2009.

Robert C. Edgar. Muscle5: High-accuracy alignment ensembles enable unbiased assessments of sequence homology and phylogeny. *Nature Communications*, 13(1): 6968, 2022.

David T. Jones, William R. Taylor, and Janet M. Thornton. The rapid generation of mutation data matrices from protein sequences. *Computer Applications in the Biosciences: CABIOS*, 8(3):275–282, 1992.

Laura Martinez Gomez, Fernando Pozo, Thomas A. Walsh, et al. The clinical importance of tandem exon duplication-derived substitutions. *Nucleic Acids Research*, 49(14):8232–8246, 2021.

Andrew Rambaut and Nicholas C. Grassly. Seq-Gen: an application for the Monte Carlo simulation of DNA sequence evolution along phylogenetic trees. *Computer Applications in the Biosciences: CABIOS*, 13(3):235–238, 1997.
